# Exceptional Response to AKT Inhibition in Patients With Breast Cancer and Germline *PTEN* Mutations

**DOI:** 10.1200/PO.19.00130

**Published:** 2019-12-05

**Authors:** Belinda Kingston, Caroline Bailleux, Suzette Delaloge, Gaia Schiavon, Veronique Scott, Magali Lacroix-Triki, T. Hedley Carr, Iwanka Kozarewa, Heidrun Gevensleben, Zoe Kemp, Alex Pearson, Nicholas Turner, Fabrice André

**Affiliations:** ^1^ Institute of Cancer Research, London, United Kingdom; ^2^ Institut Gustave Roussy, Villejuif, France; ^3^ AstraZeneca, IMED Oncology, Cambridge, United Kingdom; ^4^ Bonn University Hospital, Bonn, Germany; ^5^ The Royal Marsden NHS Foundation Trust, London, United Kingdom

## INTRODUCTION

Cowden syndrome is an autosomal dominant genetic disease with an estimated incidence of one in 200,000. Affected individuals develop multiple systemic hamartomas and have a cumulative lifetime risk of breast cancer of 85%.^1^ Approximately 80% of patients with Cowden syndrome have a germline inactivating mutation in *PTEN* (10q23.3).^2^ *PTEN* acts as a tumor suppressor gene via numerous mechanisms,^3^ one of which is antagonizing the PI3K/AKT/mTOR signaling pathway by dephosphorylating phosphatidylinositol (3,4,5)-trisphosphate (PIP3). PIP3 functions as a secondary messenger in the PI3K pathway that binds and activates proteins that have a pleckstrin homology domain, such as AKT1, and triggers their activation and localization to the plasma membrane, promoting cellular proliferation and survival.^4^

Germline *PTEN* loss-of-function mutations may result in dominant AKT activation as a driving oncogenic event in Cowden-related breast tumors.^5^ Preclinical evidence suggests that cancers with AKT activation have increased sensitivity to AKT inhibition.^6^ Preliminary clinical evidence is derived from phase I and II trials in patients with breast cancers bearing somatic mutations in the PI3K/AKT/mTOR pathway.^7-11^

Capivasertib (AZD5363, AstraZeneca) is a potent and selective oral inhibitor of all three isoforms of the serine/threonine kinase AKT (ie, AKT1, 2, 3) and has preclinical evidence of efficacy as monotherapy or in combination with cytotoxic and targeted therapies.^12-15^ Despite the encouraging progression-free survival observed with capivasertib monotherapy in heavily pretreated patients with *AKT1* E17K–mutant breast and gynecologic cancers,^9^ RECIST response rates in phase I studies only reached 22% (Table 1).^8,9,16^ Because *PTEN* loss activates AKT1, we hypothesized that tumors from patients with Cowden syndrome could be sensitive to this drug family.

**TABLE 1.**
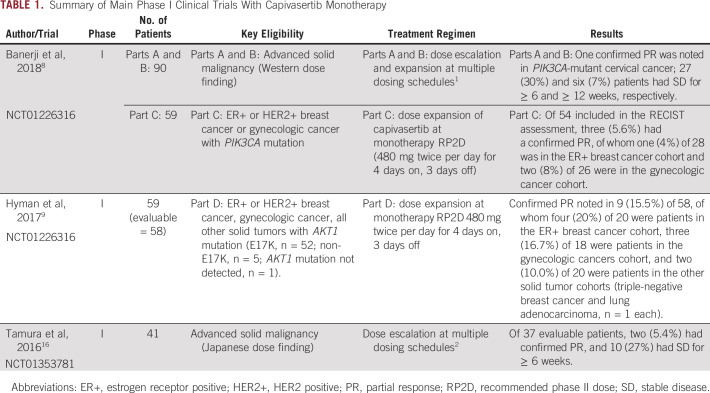
Summary of Main Phase I Clinical Trials With Capivasertib Monotherapy

### Case 1: SAFIR02 Trial

A 50-year-old woman with a family history of Cowden syndrome was diagnosed with a T3N3 breast cancer, which was estrogen (ER) and progesterone receptor negative, human epidermal growth factor receptor 2 (HER2) negative, and was designated grade III invasive carcinoma of no special type (NST). The patient received six cycles of neoadjuvant cyclophosphamide 600 mg/m^2^, epirubicin 75 mg/m^2^, and docetaxel 100 mg/m^2^ before a mastectomy with left axillary lymph node dissection (revealing residual disease in 10 of 18 lymph nodes) and adjuvant radiotherapy.

**FIG 1.**
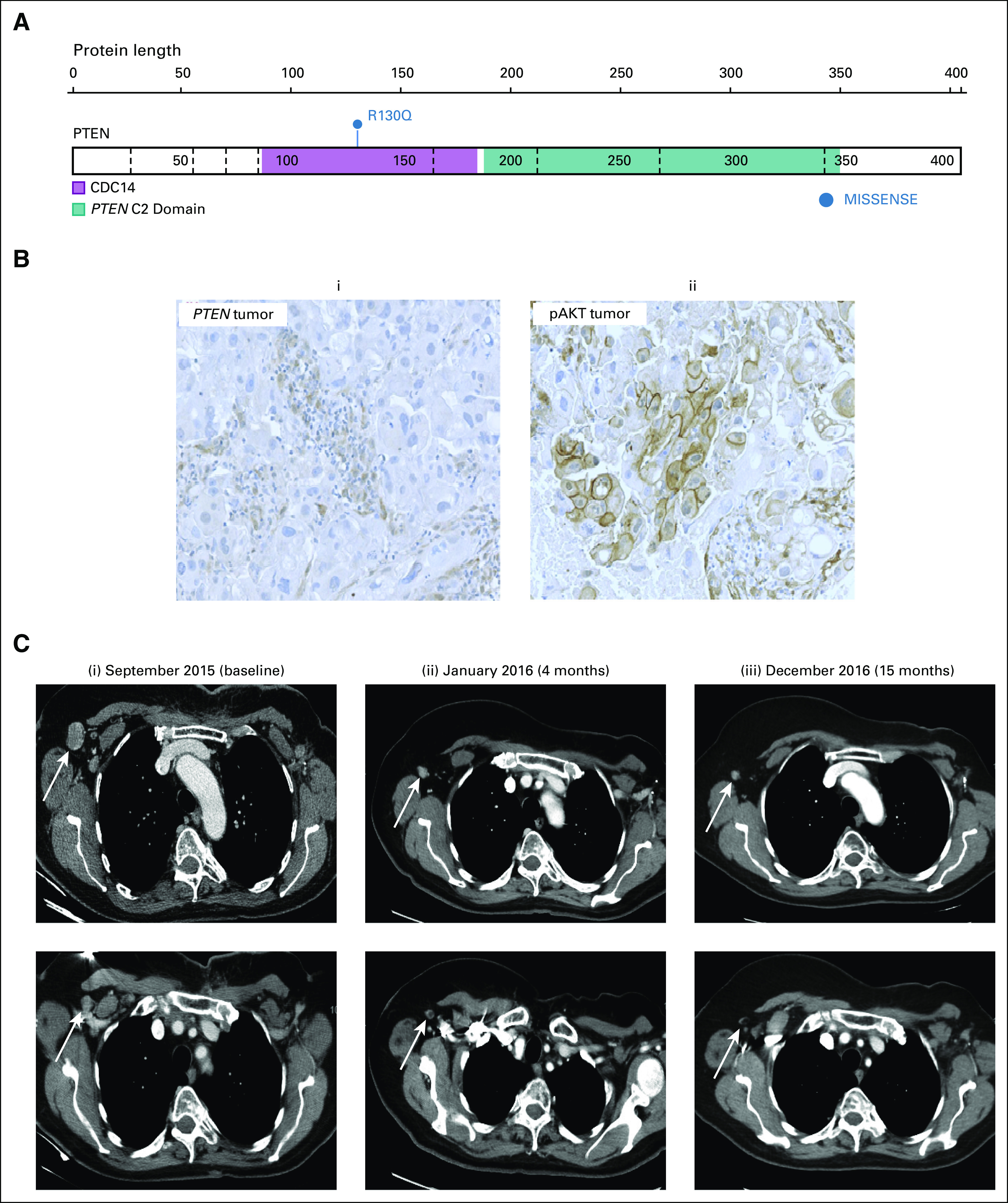
Exceptional response in patient with germline *PTEN* R130Q mutation. (A) Germline *PTEN* mutation c.389G>A, pR130Q. CDC14, phosphatase domain. Illustration from https://proteinpaint.stjude.org. (B) Immunohistochemistry demonstrating (i) absent PTEN staining in the tumor and (ii) cytoplasmic and membranous expression of pAKT in the 40% of tumor cells. (C) Computed tomography (CT) scans during the patient’s time on capivasertib, with white arrows indicating axillary disease: (i), September 2015, baseline CT showing two areas of axillary lymphadenopathy; (ii) January 2016, CT scans demonstrating partial response following 4 months of carboplatin-paclitaxel-bevacizumab chemotherapy; and (iii) December 2016, CT scans following 11 months of capivasertib monotherapy demonstrating persistent complete response before progression in February 2017.

Eight months later, the patient experienced relapse with cutaneous disease and thoracic nodal involvement. After enrolling in SAFIR02 (ClinicalTrials.gov identifier: NCT02299999), targeted panel sequencing (Ion Torrent PGM; Thermo Fisher Scientific, Villebon, France) of a fresh tumor biopsy sample revealed the presence of a heterozygous germline *PTEN* mutation (c.389G>A, p.R130Q, SNP rs121909229) alongside other variants (Fig 1A; Data Supplement). Immunohistochemistry revealed lack of *PTEN* expression in the tumor (Fig 1B). The patient received six cycles of paclitaxel (90 mg/m^2^ on days 1, 8, and 15 of a 28-day cycle) with bevacizumab (10 mg/m^2^ on days 1 and 15) and carboplatin (AUC2, days 1, 8, and 15 of a 28-day cycle, ceased after 6 weeks).

Upon completion of chemotherapy, tumor evaluation demonstrated a partial response (by RECIST, version 1.1) with 60% reduction of target lesions (Fig 1C). In the context of SAFIR02, the patient was randomly assigned to maintenance targeted therapy and received capivasertib 480 mg twice per day, 4 days on and 3 days off. This treatment was well tolerated, with no grade 2 or greater toxicities. After 3 months of capivasertib monotherapy, a complete response was observed, which was maintained for 12 months before the patient experienced progression while on capivasertib.

### Case 2: BEECH Trial

**FIG 2.**
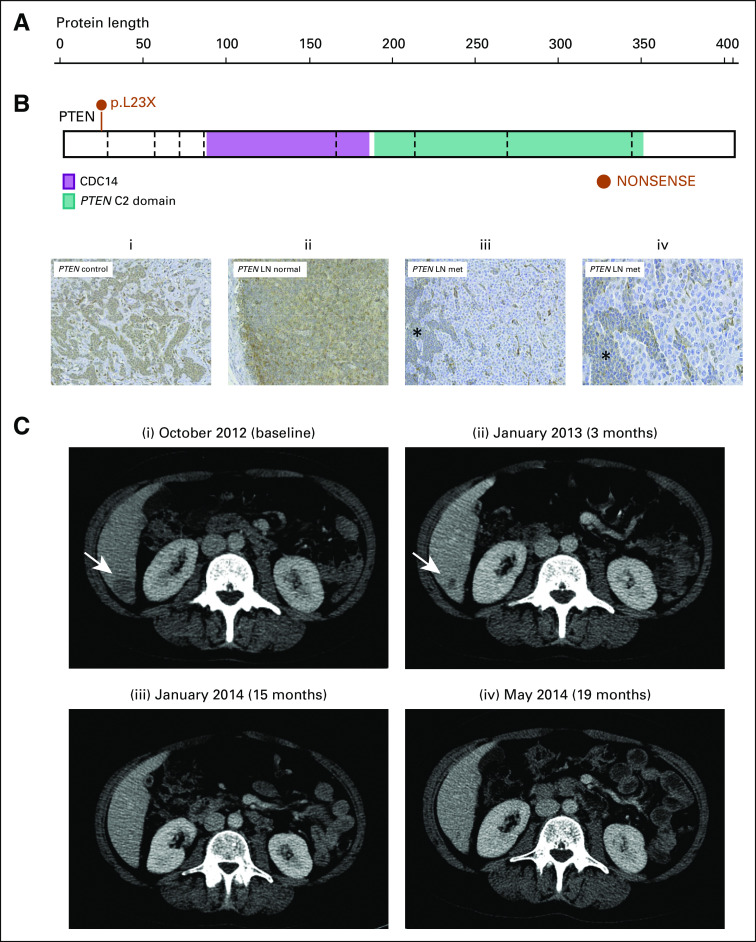
Exceptional response in patient with germline *PTEN* L23X mutation. (A) Germline *PTEN* mutation c.T68G:p.L23X. CDC14, phosphatase domain. Illustration from https://proteinpaint.stjude.org. (B) *PTEN* immunohistochemistry from (i) control tissue and (ii) noncancerous and (iii and iv) tumor-containing lymph node. The control tissue shows mainly cytoplasmic expression of *PTEN*. The noncancerous lymph node and the residual lymphatic tissue in the lymph node metastasis also show a *PTEN* expression comparable with the control. The tumor cells in the lymph node metastasis display weaker *PTEN* staining, which is mainly nucleolar. (C) Computed tomography (CT) scans during the patient’s time on capivasertib, with white arrows indicating disease in the liver: (i) October 2012, baseline CT demonstrating a liver deposit; (ii) January 2013, CT following two cycles of paclitaxel and capivasertib; (iii) CT demonstrating continued complete response on maintenance capivasertib 7 months after cessation of paclitaxel; and (iv) May 2014, final CT before progression June 2014.

In March 2010, a 37-year-old woman with known Cowden syndrome and a history of a neck arteriovenous malformation, multinodular goiter, and rectal hamartomatous polyps was diagnosed with bilateral breast cancer. On the right side, she presented with a T3, ER-positive and progesterone receptor–positive, HER2-negative, grade II invasive carcinoma NST with 20 of 23 involved lymph nodes. On the left, she presented with a 4-mm, grade II invasive carcinoma NST, strongly ER positive and HER2 negative. After a bilateral mastectomy, exome sequencing (Hiseq2500, Illumina, Cambridge, UK) of the right breast cancer and germline DNA revealed the germline *PTEN* mutation (c.T68G:p.L23X), and a second-hit somatic stop-gain *PTEN* mutation (exon 1, c.T264A:p.Y88X) with an allele frequency (AF) of 25.5%, alongside other variants (Fig 2A; Data Supplement). *PTEN* immunohistochemistry revealed reduced *PTEN* staining in the tumor (Fig 2B). Postoperative staging revealed metastatic disease with mediastinal lymph node and lung metastases. In May 2010, the patient received six cycles of chemotherapy every 3 weeks (fluorouracil 600 mg/m^2^, epirubicin 75 mg/m^2^, and cyclophosphamide 600 mg/m^2^) before starting maintenance tamoxifen. In October 2011, she underwent a bilateral salpingo-oophorectomy.

After a 28-month progression-free period, the patient had new liver metastases. In November 2012, she enrolled in the phase I/II BEECH study (ClinicalTrials.gov identifier: NCT01625286) and was assigned to receive paclitaxel plus capivasertib (part A, schedule 2). She received eight cycles of paclitaxel (90 mg/m^2^ on days 1, 8, and 15 of a 28-day cycle) combined with capivasertib (360 mg twice per day on days 2-5, 9-12, and 16-18 of each 28-day cycle). In June 2013, a computed tomography scan showed a complete response of the liver metastases. The patient continued maintenance capivasertib alone with no grade 2 or greater toxicities. She had a confirmed maintained response in May 2014 until progression occurred in June 2014—a progression-free survival of 19 months—and a maintained complete response for 12 months on capivasertib alone (Fig 2C). Plasma circulating tumor DNA analysis from baseline and progression time points during the BEECH study showed no major changes (Data Supplement).

## DISCUSSION

The AKT inhibitor capivasertib has been examined in early-phase trials (Table 1), and no complete responses have been noted, yet both patients with Cowden syndrome presented here had durable complete responses to capivasertib. Such outlier sensitivity likely reflects the germline, and therefore fundamentally clonal, nature of *PTEN* alteration. In vivo mice models with *PTEN* homozygous deletion have shown dramatic regression of the Cowden phenotype features of trichilemmomas on treatment with the mTOR (downstream from AKT) inhibitor rapamycin.^17^ In humans, Hyman et al^9^ demonstrated that tumor response to targeted treatment with capivasertib was proportional to *AKT1* mutation clonality. Furthermore, three case reports in pediatric patients and a pilot study (n = 18) in adults with *PTEN* aberrations have shown regression of phenotypic changes associated with *PTEN* loss after treatment with mTOR inhibitor sirolimus,^18-21^ demonstrating sensitivity of even nonmalignant cells carrying a germline *PTEN* mutation to PI3K-pathway inhibition. Similar to the cases presented here, no severe toxicity was noted in the pilot study,^21^ with just one patient experiencing a grade 3 adverse event (hypophosphatemia/hypercholesterolemia).

In recent years, drugs targeting the PI3K/AKT/mTOR pathway have been developed. Trials have attempted to identify molecular predictors of response by identifying alterations in the PI3K/AKT/mTOR pathway (Data Supplement). Tumor *PIK3CA* mutations are not clear predictors of response to mTOR inhibition. In contrast, AKT inhibitors in combination with paclitaxel have been shown to be more active in patients with triple-negative breast cancers harboring a *PIK3CA/PTEN/AKT1* pathway alteration.^7,10^ Similarly, PI3K inhibitors have demonstrated activity in patients with *PIK3CA* mutations.^22,23^

Of note in case 2 is the presence of the second-hit *PTEN* stop-gain mutation Y88X, present with an AF of 25.5%. However, the presence of a *TP53* mutation at an AF of 51.9% indicates that this second-hit mutation is subclonal rather than a truncal driver mutation. Conversely, the patient in case 1 does not have a *PTEN* second-hit mutation or loss of heterozygosity (LOH) yet demonstrates lack of PTEN expression. Low tumor purity can make LOH difficult to detect, and, although the estimated purity was 40%, the true purity may have been lower. Explanations for the PTEN phenotype in case 1 include undetected LOH, PTEN promotor hypermethylation,^24,25^ complex *PTEN* genomic rearrangements,^26^ and post-translational modification.^27^

Contrary to the two-hit model by Knudson et al^28^ of tumorigenesis in tumor suppressor genes, *PTEN* aberrations appear to be protumorigenic in the absence of a second hit. In 2010, Alimonti et al^29^ demonstrated that PTEN hypermorphic mice (with 80% of the normal PTEN protein level) had a greater propensity to tumorigenesis than mice with two functional alleles but were less tumorigenic than *PTEN* heterozygous mice, supporting a haploinsufficiency model of tumorigenesis in *PTEN* aberrations.

A later in vivo study of *PTEN* knock-in mouse models suggested that the conformation of PTEN underlies the dominant-negative behavior of *PTEN* heterozygous mutants. Papa et al^30^ demonstrated that PTEN is catalytically active in PIP3 dephosphorylation and subsequent downstream PI3K/AKT/mTOR pathway regulation, after dimerization. Significantly, mutant PTEN protein was able to dimerize with wild-type PTEN, but the resultant hetero-dimers were less able to hydrolyze PIP3. Moreover, mutant PTEN outcompeted and displaced wild-type PTEN protein in dimerization and membrane localization. This supports the rationale that *PTEN* heterozygous mutants act in a dominant-negative manner to promote tumorigenesis.

The patients in the cases presented here were treated with combination chemotherapy and capivasertib followed by capivasertib monotherapy. The patient in case 1 had previously demonstrated tumor resistance to taxane therapy, with residual disease after neoadjuvant chemotherapy and a short progression-free survival after treatment of primary breast cancer. The second patient achieved a complete response with the combination of paclitaxel and capivasertib and maintained this complete response for a period of 12 months on capivasertib alone, suggesting that capivasertib was highly active in this patient.

In summary, these two patients with breast cancer and different germline *PTEN* mutations both showed a dramatic response to capivasertib superior to that seen in early trials of the drug. The excellent response in these two patients, despite their differing histology, is a promising indication that targeted AKT therapy is an effective approach in patients with germline *PTEN* mutations.

## References

[1] Tan MH Mester JL Ngeow J et al Lifetime cancer risks in individuals with germline *PTEN* mutations Clin Cancer Res 18 400 407 2012 22252256 10.1158/1078-0432.CCR-11-2283 PMC3261579

[2] Eng C Will the real Cowden syndrome please stand up: Revised diagnostic criteria J Med Genet 37 828 830 2000 11073535 10.1136/jmg.37.11.828 PMC1734465

[3] Lee YR Chen M Pandolfi PP The functions and regulation of the *PTEN* tumour suppressor: New modes and prospects Nat Rev Mol Cell Biol 19 547 562 2018 29858604 10.1038/s41580-018-0015-0

[4] Miller TW Pérez-Torres M Narasanna A et al Loss of phosphatase and tensin homologue deleted on chromosome 10 engages *ErbB3* and insulin-like growth factor-I receptor signaling to promote antiestrogen resistance in breast cancer Cancer Res 69 4192 4201 2009 19435893 10.1158/0008-5472.CAN-09-0042 PMC2724871

[5] Altomare DA Testa JR Perturbations of the AKT signaling pathway in human cancer Oncogene 24 7455 7464 2005 16288292 10.1038/sj.onc.1209085

[6] Davies BR Guan N Logie A et al Tumors with *AKT1E17K* mutations are rational targets for single agent or combination therapy with AKT inhibitors Mol Cancer Ther 14 2441 2451 2015 26351323 10.1158/1535-7163.MCT-15-0230

[7] Kim SB Dent R Im SA et al Ipatasertib plus paclitaxel versus placebo plus paclitaxel as first-line therapy for metastatic triple-negative breast cancer (LOTUS): A multicentre, randomised, double-blind, placebo-controlled, phase 2 trial Lancet Oncol 18 1360 1372 2017 28800861 10.1016/S1470-2045(17)30450-3 PMC5626630

[8] Banerji U Dean EJ Pérez-Fidalgo JA et al A phase I open-label study to identify a dosing regimen of the pan-AKT inhibitor AZD5363 for evaluation in solid tumors and in *PIK3CA*-mutated breast and gynecologic cancers Clin Cancer Res 24 2050 2059 2018 29066505 10.1158/1078-0432.CCR-17-2260

[9] Hyman DM Smyth LM Donoghue MTA et al AKT inhibition in solid tumors with *AKT1* mutations J Clin Oncol 35 2251 2259 2017 28489509 10.1200/JCO.2017.73.0143 PMC5501365

[10] Schmid P Abraham J Chan S et al AZD5363 plus paclitaxel versus placebo plus paclitaxel as first-line therapy for metastatic triple-negative breast cancer (PAKT): A randomised, double-blind, placebo-controlled, phase II trial J Clin Oncol 36 2018 (suppl; abstr 1007) 10.1200/JCO.2018.36.15_suppl.1007 31841354

[11] Smyth L Oliveira M Ciruelos E et al AZD5363 in combination with fulvestrant in AKT1-mutant ER-positive metastatic breast cancer Cancer Res 78, 2018 (suppl; abstr P5-21-32)

[12] Patnaik A Appleman LJ Tolcher AW et al First-in-human phase I study of copanlisib (BAY 80-6946), an intravenous pan-class I phosphatidylinositol 3-kinase inhibitor, in patients with advanced solid tumors and non-Hodgkin’s lymphomas Ann Oncol 27 1928 1940 2016 27672108 10.1093/annonc/mdw282 PMC5035790

[13] Zhang C Xu B Liu P Addition of the p110α inhibitor BYL719 overcomes targeted therapy resistance in cells from Her2-positive-PTEN-loss breast cancer Tumour Biol 37 14831 14839 2016 27639383 10.1007/s13277-016-5381-7

[14] Yu Y Savage RE Eathiraj S et al Targeting AKT1-E17K and the PI3K/AKT pathway with an allosteric AKT inhibitor, ARQ 092 PLoS One 10 e0140479 2015 26469692 10.1371/journal.pone.0140479 PMC4607407

[15] Agarwal R Liebe S Turski ML et al Targeted therapy for genetic cancer syndromes: Von Hippel-Lindau disease, Cowden syndrome, and Proteus syndrome Discov Med 19 109 116 2015 25725225

[16] Tamura K Hashimoto J Tanabe Y et al Safety and tolerability of AZD5363 in Japanese patients with advanced solid tumors Cancer Chemother Pharmacol 77 787 795 2016 26931343 10.1007/s00280-016-2987-9 PMC4819940

[17] Squarize CH Castilho RM Gutkind JS Chemoprevention and treatment of experimental Cowden’s disease by mTOR inhibition with rapamycin Cancer Res 68 7066 7072 2008 18757421 10.1158/0008-5472.CAN-08-0922

[18] Marsh DJ Trahair TN Martin JL et al Rapamycin treatment for a child with germline *PTEN* mutation Nat Clin Pract Oncol 5 357 361 2008 18431376 10.1038/ncponc1112

[19] Schmid GL Kässner F Uhlig HH et al Sirolimus treatment of severe *PTEN* hamartoma tumor syndrome: Case report and in vitro studies Pediatr Res 75 527 534 2014 24366516 10.1038/pr.2013.246

[20] Iacobas I Burrows PE Adams DM et al Oral rapamycin in the treatment of patients with hamartoma syndromes and *PTEN* mutation Pediatr Blood Cancer 57 321 323 2011 21360661 10.1002/pbc.23098

[21] Komiya T Blumenthal GM Ballas MS et al A pilot study of sirolimus (S) in subjects with Cowden syndrome (CS) with germ-line mutations in *PTEN* J Clin Oncol 31 2532-2532 2013 31350329 10.1634/theoncologist.2019-0514 PMC6975943

[22] Juric D Ciruelos E Rubovszky G et al Alpelisib + fulvestrant for advanced breast cancer: Subgroup analyses from the phase III SOLAR-1 trial Cancer Res 79, 2019 (suppl; abstr GS3-08)

[23] Baselga J Dent SF Cortés J et al Phase III study of taselisib (GDC-0032) + fulvestrant (FULV) v FULV in patients (pts) with estrogen receptor (ER)-positive, *PIK3CA*-mutant (MUT), locally advanced or metastatic breast cancer (MBC): Primary analysis from SANDPIPER J Clin Oncol 36 2018 (suppl; abstr LBA1006) 10.1200/JCO.2018.36.18_suppl.LBA1006

[24] Luo S Chen J Mo X The association of *PTEN* hypermethylation and breast cancer: A meta-analysis OncoTargets Ther 9 5643 5650 2016 10.2147/OTT.S111684 PMC5026181 27672335

[25] Zhang H-Y Liang F Jia Z-L et al *PTEN* mutation, methylation and expression in breast cancer patients Oncol Lett 6 161 168 2013 23946797 10.3892/ol.2013.1331 PMC3742525

[26] Jones N Bonnet F Sfar S et al Comprehensive analysis of *PTEN* status in breast carcinomas Int J Cancer 133 323 334 2013 23319441 10.1002/ijc.28021

[27] Song MS Salmena L Pandolfi PP The functions and regulation of the *PTEN* tumour suppressor Nat Rev Mol Cell Biol 13 283 296 2012 22473468 10.1038/nrm3330

[28] Knudson AG Two genetic hits (more or less) to cancer Nat Rev Cancer 1 157 162 2001 11905807 10.1038/35101031

[29] Alimonti A Carracedo A Clohessy JG et al Subtle variations in *PTEN* dose determine cancer susceptibility Nat Genet 42 454 458 2010 20400965 10.1038/ng.556 PMC3118559

[30] Papa A Wan L Bonora M et al Cancer-associated *PTEN* mutants act in a dominant-negative manner to suppress PTEN protein function Cell 157 595 610 2014 24766807 10.1016/j.cell.2014.03.027 PMC4098792

